# Are estimates of wind characteristics based on measurements with Pitot tubes and GNSS receivers mounted on consumer-grade unmanned aerial vehicles applicable in meteorological studies?

**DOI:** 10.1007/s10661-017-6141-x

**Published:** 2017-08-03

**Authors:** Tomasz Niedzielski, Carsten Skjøth, Małgorzata Werner, Waldemar Spallek, Matylda Witek, Tymoteusz Sawiński, Anetta Drzeniecka-Osiadacz, Magdalena Korzystka-Muskała, Piotr Muskała, Piotr Modzel, Jakub Guzikowski, Maciej Kryza

**Affiliations:** 1 Institute of Geography and Regional Development, Faculty of Earth Sciences and Environmental Management, University of Wrocław, pl. Uniwersytecki 1, 50-137 Wrocław, Poland; 2 Institute of Science and the Environment, University of Worcester, Henwick Grove, Worcester, WR2 6AJ UK

**Keywords:** Unmanned aerial vehicle, Wind measurement, Pitot tube, GNSS, Izera Mountains, Poland

## Abstract

The objective of this paper is to empirically show that estimates of wind speed and wind direction based on measurements carried out using the Pitot tubes and GNSS receivers, mounted on consumer-grade unmanned aerial vehicles (UAVs), may accurately approximate true wind parameters. The motivation for the study is that a growing number of commercial and scientific UAV operations may soon become a new source of data on wind speed and wind direction, with unprecedented spatial and temporal resolution. The feasibility study was carried out within an isolated mountain meadow of Polana Izerska located in the Izera Mountains (SW Poland) during an experiment which aimed to compare wind characteristics measured by several instruments: three UAVs (swinglet CAM, eBee, Maja) equipped with the Pitot tubes and GNSS receivers, wind speed and direction meters mounted at 2.5 and 10 m (mast), conventional weather station and vertical sodar. The three UAVs performed seven missions along spiral-like trajectories, most reaching 130 m above take-off location. The estimates of wind speed and wind direction were found to agree between UAVs. The time series of wind speed measured at 10 m were extrapolated to flight altitudes recorded at a given time so that a comparison was made feasible. It was found that the wind speed estimates provided by the UAVs on a basis of the Pitot tube/GNSS data are in agreement with measurements carried out using dedicated meteorological instruments. The discrepancies were recorded in the first and last phases of UAV flights.

## Introduction

Numerous techniques utilized in environmental monitoring make use of unmanned aerial vehicles (UAVs), commonly known as drones, which enable to carry out aerial observations of terrain using a wide range of cameras (e.g., Colomina and Molina 2014) as well as to conduct measurements of the air (e.g., Martin et al. 2011). Particularly common on the market are photogrammetric consumer-grade UAVs, which are targeted at acquiring oblique aerial images. If a pre-defined longitudinal and lateral overlap is high enough, the photographs may be processed using the Structure-from-Motion (SfM) algorithms and, consequently, can be utilized to produce digital surface models (DSMs) and orthophotomaps (Westoby et al. 2012). Apart from dedicated photogrammetric solutions, there are numerous commercial-grade UAVs that are designed to take photographs, make movies or carry out other tasks such as for instance search and rescue activities (Goodrich et al. 2008), transportation of goods to victims (Bernard et al. 2011) and commercial transportation (Murray and Chu 2015). A growing number of commercial and scientific applications of drones as well as the use of such UAVs by non-professional enthusiasts lead to a frequent usage of aerospace in its lower layer of the troposphere. Most UAVs are equipped with the Pitot tube—a pressure measurement instrument which is commonly used to determine local flow velocity and therefore, in the context of aircraft, its airspeed—and the GNSS (Global Navigation Satellite System) receiver. The two devices are routinely used for navigating a UAV, and are often utilized to calculate wind speed and wind direction (Van den Kroonenberg et al. 2008; Cho et al. 2011; Langelaan et al. 2011). Along with a growing popularity and usage of UAVs, such data on wind characteristics may soon become valuable source of information on air dynamics in lower troposphere, with the unprecedented spatial and temporal resolution. However, the quality of these data may be disputable, especially in the mountains, and therefore the question arises as to whether they may be applicable in meteorological studies, such as for instance numerical weather predictions (NWPs) or dispersion of atmospheric pollutants, e.g., emitted from forest fires. The practical usefulness of such data—acquired on the occasion of, for instance, photogrammetric flights—goes beyond NWPs (Jonassen et al. 2012) and covers numerous applications, including real-time weather monitoring in ungauged areas. In particular, such wind estimates may be utilized in experiments that make a concurrent use of aerial photography and weather monitoring, such as for instance snow water equivalent determination in near real time (Elder et al. 1998; Bühler et al. 2016; Miziński and Niedzielski 2017).

Intrinsically, there are several meteorological instruments that are dedicated to measure wind speed and wind direction. They include wind speed and wind direction meters installed conventionally at a level of approximately 2 m above the ground (often in the vicinity of the Stevenson screen) or at greater heights above the ground (often mounted on meteorological masts). Less convectional instruments are sodars (wind profilers), with their vertical and horizontal versions, which measure scattering of sound waves to determine wind speed at different levels. Weather balloons can also serve the purpose of acquiring data on wind characteristics; however, they are usually utilized to monitor high altitudes. Manned aircrafts and recently UAVs can host professional wind speed and wind direction sensors, and measure these parameters at various heights. Although these professional meteorological equipment offer reliable data on wind speed and wind direction, these instruments, even if integrated within a larger network, cannot guarantee a dense spatial coverage which, for instance, is very important to improve the NWP skills (Jonassen et al. 2012). Indeed, wind speed and wind direction meters, mounted either at 2 m or on masts, as well as sodars and weather balloons produce pointwise data. Manned aircrafts and meteorological UAVs acquire spatial data; however, they are limited in time to a particular research mission. Therefore, there is a need to seek new possibilities that may ensure better spatial and temporal coverage of wind speed and wind direction measurements. This may be achieved using the non-meteorological UAVs which, as discussed above, become very common and for their navigation use wind measurements obtained from the Pitot tube readings and the GNSS observations.

Langelaan et al. (2011) explicitly identified the approach in question as a low-cost method for atmospheric measurement and sampling system. Although there were several attempts to verify the UAV-based wind estimates against meteorological observations (Van den Kroonenberg et al. 2008) and simulations (Langelaan et al. 2011), they usually focused on lowland terrain. In addition, the most common experimental setup assumed the use of one UAV. Therefore, there is a need for investigating the usefulness of the aforementioned approach in remote mountainous environments usually with sparse meteorological measurements and for providing evidences of reproducibility, i.e., that various consumer-grade UAVs may offer wind estimates of similar accuracies. In order to conduct such an experimental verification, the measurement campaign has been set up by the University of Wrocław (Poland) and the University of Worcester (UK). The campaign was organized in the Izera Mountains (southwestern Poland) and supported by Świeradów Forest Inspectorate (Poland). Three different UAVs were used to estimate wind speed and wind direction over an isolated mountain meadow surrounded by dense conifer forest. The estimates were possible to be compared with measurements carried out using wind speed and wind direction meters mounted at 2.5 and 10 m (mast), conventional weather station and vertical sodar. The objective of this paper is therefore to report on the results of comparisons of wind estimates: (1) between three UAVs (reproducibility test) and (2) between individual UAVs and terrestrial meteorological instruments (validation test). The research hypothesis, which is supposed to be verified in this paper, reads as follows: “estimates of wind speed and wind direction based on the Pitot tube measurements and the GNSS observations carried out jointly by consumer-grade UAVs are consistent with values of these parameters recorded by professional meteorological instruments”.

## Study area and synoptic situation

### Terrain characteristics

**Fig. 1:**
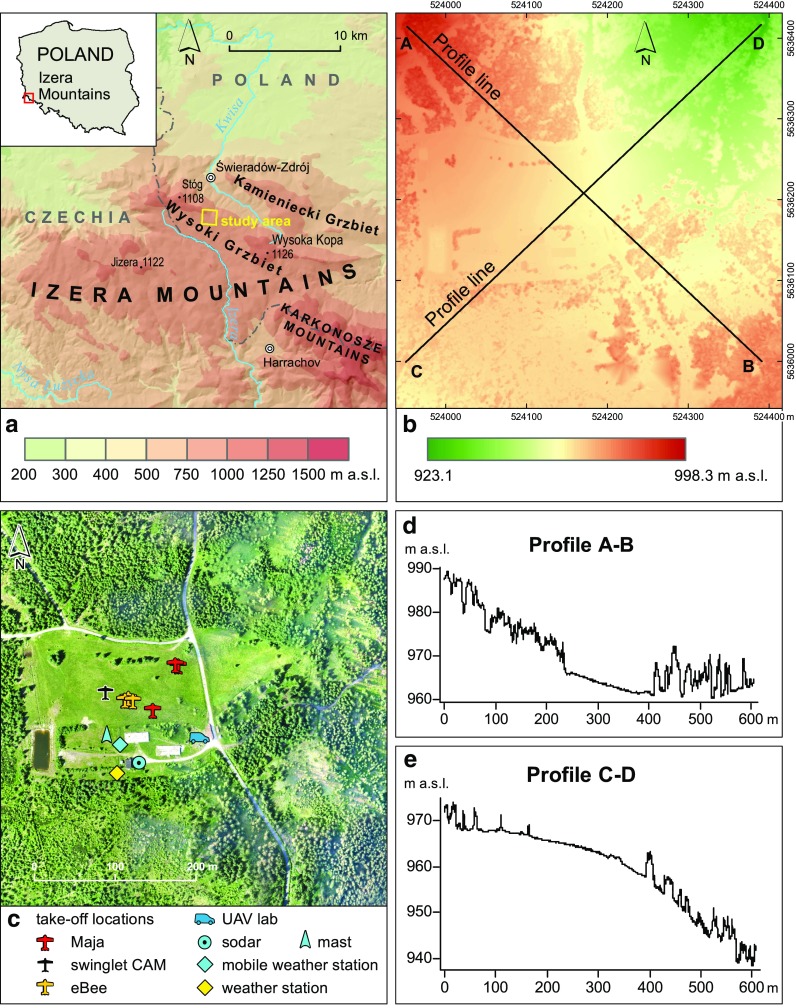
Location of the study area (**a**), visualization of digital surface model of the study area along with two profile lines superimposed (**b**), orthophotomap of the study area with locations of devices used to carry out measurements (**c**), vertical terrain profile along profile line A–B (**d**), vertical terrain profile along profile line C–D (**e**). Maps and profiles produced using geographic information system (GIS) tools

The measurement campaign, during which UAVs and terrestrial meteorological instruments were utilized, was carried out in the central part of the Izera Mountains that belong to the western part of the Sudetes Mountains, stretching along Polish-Czech border. The border divides the Izera Mountains into two parts, with the higher one belonging to Poland. In the Polish part, there are two main parallel ridges: the Kamienicki Grzbiet ridge (lower) in the north and the Wysoki Grzbiet ridge (higher) in the south. The highest peak of the Izera Mountains is Mt. Wysoka Kopa (1126 m a.s.l.). These two ridges are horsts, and are separated by deep single graben structure used by the Kwisa river (Fig. 1a).

The research area is situated in the west part of the Wysoki Grzbiet ridge, in the pass between Mt. Świeradowiec (1037 m a.s.l.) and Mt. Podmokła (1001 m a.s.l.). The area includes a mid-forest glade with individual trees, called Polana Izerska, and its forested surroundings. Polana Izerska is an isolated rectangular meadow of approximate dimensions 250 × 170 m, within which elevations vary between approximately 951 and 976 m a.s.l. (Fig. 1b). The spruces form the surrounding forest (Fig. 1c) in which trees are of average heights, up to 15 m Polana Izerska is locally peaty and marshy. In the western part of the meadow, there are two small water reservoirs.

The research area is morphologically diverse. In the south, within a fragment of the mountain pass between Mt. Świeradowiec and Mt. Podmokła, the terrain is nearly flat. The altitude differences are small and reach only 10–15 m (Fig. 1d). The north part is located on the south slopes of Mt. Świeradowiec which continue eastward and northeastward to the deep valley of the Mokrzyca stream. In this part, the range of altitudes reaches 50 m (Fig. 1e). The considerable slopes that occur in the contact with relatively flat terrain (Fig. 1b) significantly affect wind field. Westerly winds prevail in the Izera Mountains.

Between 7 and 10 July 2015, two universities, the University of Wrocław (Poland) and the University of Worcester (UK), carried out joint UAV workshop, the aim of which was to use UAVs for monitoring atmospheric phenomena. One of the main objectives was to measure wind characteristics using drones, with a particular emphasis put on consumer-grade UAVs. Wind speed and wind direction estimates based on drones were possible to be verified against measurements. Field measurements of wind characteristics were carried out on 7 and 8 July 2015.

### Synoptic situation on 7–8 July 2015

Sea level pressure maps published by the British Met Office (available at www.wetterzentrale.de) were used for the analysis of synoptic conditions. In addition, data obtained during meteorological measurement campaign in Polana Izerska as well as hourly SYNOP reports from three meteorological stations located in the vicinity of study area: Liberec (WMO code 11603, coordinates 50° 46′ N 15° 01′ E, altitude 398 m a.s.l., distance to Polana Izerska 24 km), Jelenia Góra (WMO code 12500, coordinates 50° 54′ N 15° 48′ E, altitude 342 m a.s.l., distance from Polana Izerska 27 km) and Śnieżka (WMO code 12510, coordinates 50° 44′ N 15° 44′ E, altitude 1603 m a.s.l., distance from Polana Izerska 32 km) were used to describe the meteorological background. Mt. Śnieżka is located in the Karkonosze Mts. (west part of them is in Fig. 1a) and is the highest peak of the entire Sudetes. The SYNOP data, acquired by the Institute of Meteorology and Water Management – National Research Institute (Instytut Meteorologii i Gospodarki Wodnej – Państwowy Instytut Badawczy; IMGW–PIB), were obtained from www.ogimet.com.

**Fig. 2:**
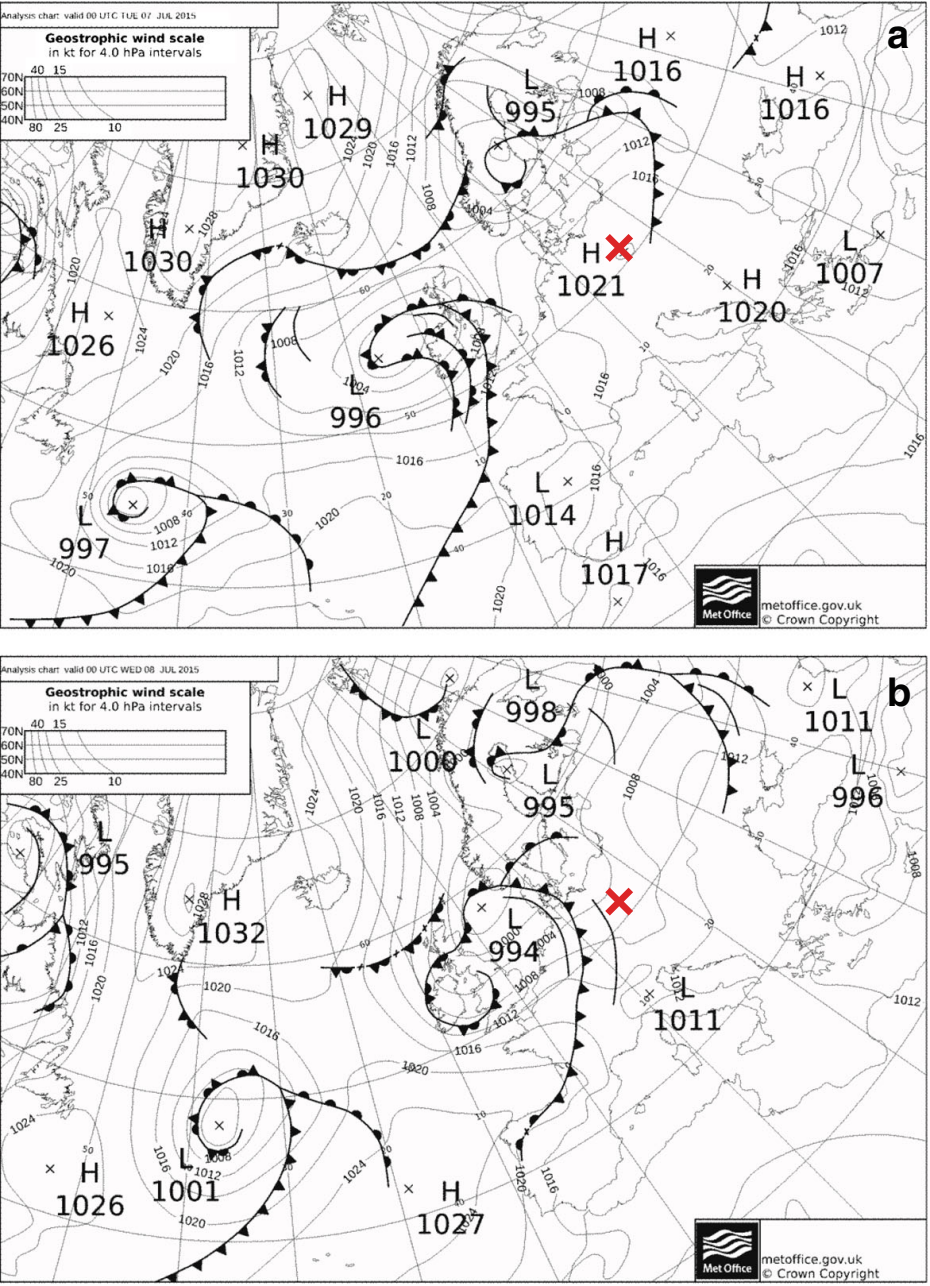
Synoptic situation over Europe on 07/07/2015 00:00 UTC (**a**) and 08/07/2015 00:00 UTC (**b**). Approximate location of the study area marked by *red X* (source of SYNOP maps: www.wettercentrale.de)

**Fig. 3:**
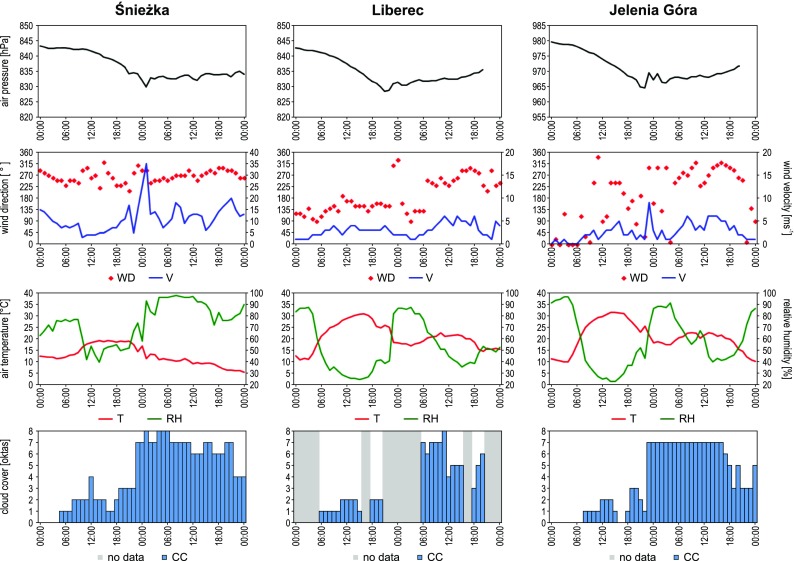
Meteorological parameters (*P*, *V*, *WD*, *T*, *RH*, and cloud cover *CC*) measured at three synoptic stations (Śnieżka, Liberec, and Jelenia Góra) from 07/07/2015 00:00 UTC to 09/07/2015 00:00 UTC (based on SYNOP reports obtained from www.ogimet.com)

On 7 July 2015, the pressure field over the study area was relatively uniform and shaped under the influence of high pressure system with weak gradient, centered southward from Poland (Fig. 2a). General direction of air advection in the study area was from the west, with slight deviation to WSW, which is confirmed by the meteorological data from Śnieżka that may represent free atmosphere (Fig. 3). Mean daily wind velocity was 8.4 ms^−1^ at Śnieżka and 2.6 and 2.0 ms^−1^, respectively, at Liberec and Jelenia Góra stations. Mean wind speed recorded at the level of 10 m a.g.l. during the measurement campaign in Polana Izerska was equal to 4.7 ms^−1^.

The synoptic situation changed on 8 July 2015, when the study area was under the influence of a low pressure system (with a center moving from the British Isles towards southern Scandinavia) and accompanying atmospheric fronts (Fig. 2b). The passing of the system manifested in a clear pressure drop (about 10 hPa), observed in all three synoptic stations (Fig. 3). General westerly direction of air advection did not change, however, deviation from the main direction moved towards WNW. Also the increase in horizontal pressure gradients (Fig. 2) caused the increase in airflow dynamics. This manifested in a clear rise of wind speed, both in the study area and at neighboring weather stations (Fig. 3)—mean daily wind speed was equal to 14.0 ms^−1^ at Śnieżka, 3.4 ms^−1^ in Liberec, and 3.3 ms^−1^ in Jelenia Góra. Mean wind speed recorded during the measurement campaign in Polana Izerska was equal to 5.6 ms^−1^.

The observed change in synoptic conditions caused an essential difference of meteorological conditions between the first and the second day of the measurement campaign. During the first day, relatively high temperature was recorded within the entire area, with the maximum temperature of 26 °C in Polana Izerska and 31, 32, and 20 °C at the nearest WMO stations, i.e., Liberec, Jelenia Góra, and Śnieżka, respectively. During the day, the cloudiness was moderate (about 25%). Convective clouds (*Cu med*, *Cu hum* and also *Cu con* in the afternoon) were predominant. At night (7/8 July 2015), a cold atmospheric front, connected with a low pressure system, passed over the study area. The front was accompanied by intensive thunderstorms and advection of cold maritime Polar air mass. As a result, the second day of the measurement campaign was much colder. Maximum air temperature in Polana Izerska was of 19 °C, and at the studied WMO stations, the maximum temperatures were equal to 23 °C (Liberec), 24 °C (Jelenia Góra), and 12 °C (Śnieżka). In the study area and its whole vicinity cloudiness reached the level of 80–100%, with predominant *Sc* clouds. In the late afternoon, near the end of the second measurement session, development of *Cu con* and *Cb* clouds was also observed.

## Data and methods

The comparison between wind speed/direction estimates obtained by UAVs and their equivalents measured by meteorological instruments is carried out in the following two exercises: comparison of the wind characteristics obtained by different drones (reproducibility test) and comparison of wind speed estimates based on UAV data with height-corrected wind speed data acquired from meteorological sensors installed on the 10-m mast (validation test).

### Wind characteristics over Polana Izerska measured by unmanned aerial vehicles

Speed and direction of the horizontal wind can be calculated indirectly, knowing the true airspeed vector (speed of a UAV in respect to the atmosphere) and the groundspeed vector (speed of a UAV in respect to the Earth’s surface). The true airspeed can be estimated from measurements carried out using the Pitot tube installed on a UAV, while the groundspeed may be computed using the readings recorded by a GNSS receiver mounted onboard a UAV. In order to estimate the airspeed vector, the Pitot tube measurements of the total pressure (sum of dynamic and static pressure) are separated into the dynamic and static components. Subsequently, the dynamic pressure is expressed as a function of air density and airspeed. Finally, the airspeed can be obtained knowing the total pressure (Pitot tube), the static pressure and air density (Barton 2012). The groundspeed vector may be computed from the GNSS velocities in north and east directions. The horizontal wind vector can be obtained by subtracting the true airspeed vector from the groundspeed vector (e.g., Van den Kroonenberg et al. 2008).

**Fig. 4:**
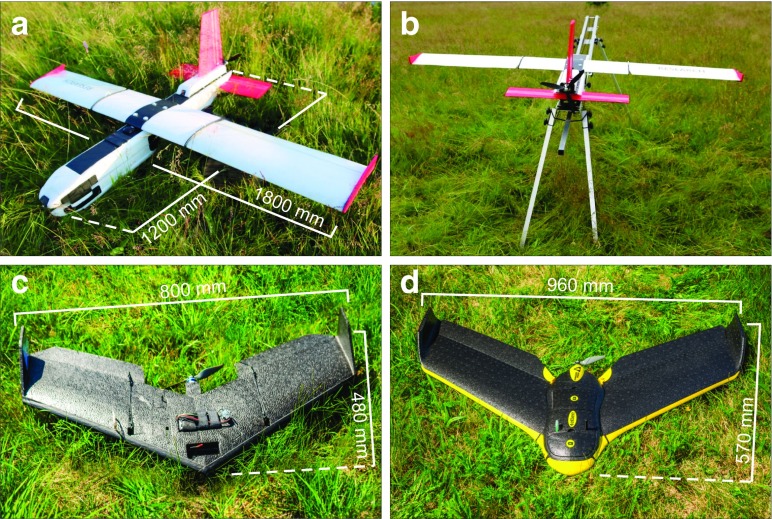
Photographs of consumer-grade UAVs used in the measurement campaign. Maja (**a**, **b**). swinglet CAM (**c**). eBee (**d**)

Three fixed-wing UAVs were used during the UAV measurement campaign in the Izera Mountains on 7–8 July 2015. The first one, known as Maja (manufacturer: Bormatec), has a wingspan of 1.80 m and its length is of 1.20 m (Fig. 4a). Maja’s weight is approximately equal to 2.5 kg. It is launched using a dedicated catapult (Fig. 4b). Maja is designed to carry out various tasks (according to the manufacturer: surveying, aerial imagery, video production, humanitarian missions, environmental and civil protection, meteorological observations) where these facilities must be implemented by the users as Bormatec only provides the frame and navigation system. This version contains a NIR camera which is a modified Canon Powershot SX260 HS. The atmospheric sensors on board detect temperature, humidity, and pressure. The Pitot tube is used to estimate airspeed which—along with GNSS readings—is used to obtain wind characteristics. The second UAV which used in the experiment is swinglet CAM (manufacturer: senseFly), an ultralight (approximately 0.7 kg) photogrammetric drone with wingspan of 0.80 m and a total length of 0.48 m (Fig. 4c). Its frame hosts a consumer-grade camera compartment (we use two cameras: Canon IXUS 220HS and Canon ELPH 300HS). Navigation of swinglet CAM is enhanced with the Pitot tube records. Wind characteristics are saved in log files which are stored in the open ASCII format. The third UAV used in the experiment is eBee, which is a commonly used consumer-grade photogrammetric drone manufactured by senseFly. The wingspan of eBee is of 0.96 m, while its length equals to 0.57 m (Fig. 4d). Weight of eBee approximately equals to 0.67 kg. Its payload is limited to a single consumer-grade camera (our setup consists of both RGB and NIR cameras of Canon S110). The Pitot tube belongs to standard devices mounted on the eBee frame and, similarly to swinglet CAM, is used to enhance navigation of the drone.

Seven flights, along the spiral-like trajectories, were performed (Table 1) due to the need of measuring changes in wind vectors, pressure and temperature with height typically found with the Ekman layer of the atmosphere (e.g., Seinfeld and Pandis 2006) and of practical navigation reasons with fixed wings UAVs. Practical navigation with fixed-wing UAVs is typically done using waypoints, often along linear paths. During strong winds, when UAVs fly along the wind, UAVs can approach a waypoint with more than 20 ms^−1^ with reference to the surface and against the wind with only 2–4 ms^−1^. Experience has shown that this causes that the UAV inadvertently passes the waypoint with a substantial distance before a full turn is completed, thereby providing less control over the flight path. Flying in circular structures overcomes this problem and provides a much more stable and predictable flight path. On 7 July 2015, swinglet CAM (flight no. 1) and eBee (flight no. 2) were used in the evening, and equal radii of the spirals (30 m) were adopted. The flight no. 1 was planned to reach the approximate above takeoff (ATO) height of 75 m, but wind resistance of swinglet CAM significantly constrained the mission and limited its time. The flight no. 2 was set to climb 130 m ATO and a bigger endurance of eBee enabled to perform the entire spiral-like mission. On 8 July 2015, Maja (flight nos. 3, 6, 7) and eBee (flight nos. 4, 5) were utilized in the morning and afternoon. Maja’s radii were set to 90 and 62 m, whereas for eBee the radii of all spirals were kept equal (30 m). The maximum planned ATO altitudes were similar for flight nos. 3–7, ranging from 115 to 130 m ATO.

**Table 1:** Basic characteristics of UAV flights targeted at real-time observations of wind speed and wind direction over Polana Izerska in the Izera Mountains (SW Poland) during the measurement campaign on 7–8 July 2015

UAV	No. flight	Radius of circular trajectory [m]	Desired maximum altitude [m ATO]	Date	Time [UTC]	Total flight time [min]
swinglet CAM	1	30	75	07/07/2015	18:23:26–18:28:59	5.55
eBee	2	30	130	07/07/2015	19:01:52–19:18:04	16.20
Maja	3	90	125	08/07/2015	07:59:51–08:18:28	18.62
eBee	4	30	130	08/07/2015	08:24:37–08:34:55	10.30
eBee	5	30	130	08/07/2015	08:38:01–08:47:56	9.92
Maja	6	90	130	08/07/2015	08:48:47–09:25:21	36.57
Maja	7	62	115	08/07/2015	09:36:22–09:50:07	13.75

Four specific flights were selected to carry out the reproducibility test, and therefore two pairs of UAV missions were investigated. Firstly, wind speed and wind direction estimates based on measurements acquired during the flight nos. 1 and 2 were compared in order to check if the use of swinglet CAM and eBee produces similar wind characteristics. The choice of the two missions was due to the fact that the flight no. 2 began approximately 30 min after accomplishing the flight no. 1, and therefore wind field was controlled by the same synoptic situation (Fig. 2a). Secondly, the wind characteristics obtained on a basis of the UAV-acquired data were compared between yet another pair of drones, namely Maja and eBee (flight nos. 3 and 4). Similarly to the swinglet–eBee case (07/07/2015), the flight no. 4 was initiated approximately 3 min after completing the flight no. 3. The short time gap ensures a relative stability of wind characteristic, in particular in the case of change of synoptic situations that occurred between 7 and 8 July 2015 (Fig. 2). In the validation test, wind estimates from three missions performed using three UAVs (flight nos. 1, 3, and 4) were used against a background of wind speed data obtained using a wind meter installed at the 10-m mast. For a sake of brevity, results obtained for flight nos. 5–7 are not presented in the paper. Basic characteristics of wind speed and wind direction recorded during flights 1–7 at the altitude of 1050 m a.s.l. are presented in Table 2.

**Table 2:** Basic statistics of wind speed and wind direction estimated for seven flights at the altitude of 1050 m a.s.l (74–99 m above Polana Izerska)

UAV	No. flight	Mean wind speed at 1050 m a.s.l. [ms^−1^]	Standard deviation of wind peed at 1050 m a.s.l. [ms^−1^]	Mean wind direction at 1050 m a.s.l. [degrees]	Standard deviation of wind direction at 1050 m a.s.l. [degrees]
swinglet CAM	1	8.9	1.7	180.9	5.9
eBee	2	11.5	0.9	179.4	3.7
Maja	3	9.5	2.5	239.9	8.7
eBee	4	8.5	0.6	244.9	9.6
eBee	5	10.0	1.2	237.1	11.2
Maja	6	11.2	0.8	231.0	3.3
Maja	7	3.6^a^	–	243.7^a^	–

^a^Only one value recorded at a level of approximately 1050 m a.s.l.

### Wind characteristics over Polana Izerska measured by meteorological instruments

During the measurement campaign in Polana Izerska, two 1-day measurement sessions for meteorological background and vertical structure of the atmosphere were held. The first session (7 July 2015) covered the time from 08:15 to 18:30 UTC (10:15–20:30 local time). The second session was held on 8 July 2015 and covered the period from 06:05 to 14:10 UTC (08:05–16:10 local time). Both sessions covered the time of measurements held with use of UAV and provided relevant reference data. The meteorological measurements were done using of two automatic weather stations MetPak Pro produced by Gill Instruments. The stations enable an automatic registration of air temperature (*T*) and humidity (*RH*) as well as air pressure (*P*). MetPak Pro stations are also equipped with an integrated acoustic anemometer that allows to measure horizontal wind velocity (*V*) and wind direction (*WD*). Measurement characteristics of all sensors built in the MetPak Pro station are shown in Table 3.

**Table 3:** Measurement characteristics of the MetPak Pro sensors (Gill Instruments 2015)

Parameter	Range	Accuracy	Resolution
Air temperature Pt100 1/3 class B	–50 to +100 °C	±0.1 °C	0.1 °C
Relative humidity	0–100% RH	±0.8% at 23 °C	0.1% RH
Air pressure	600–1100 hPa	±0.5 hPa	0.1 hPa
Wind velocity (acoustic measurement)	0–60 ms^−1^	±2% at 12 ms^−1^	0.01 ms^−1^
Wind direction (acoustic measurement)	0–359°	±3° at 12 ms^−1^	1°

Both stations were located in the southwestern part of Polana Izerska, in a field covered by grass with the distance of approximately 30 m to the nearest trees, the height of which did not exceed 5 m. The first weather station was placed at a height of 2.5 m a.g.l., while the second one was placed at the top of a mast (10 m a.g.l). The detailed locations of both the stations are presented in Fig. 1c. Time resolution of all meteorological measurements (*T*, *RH*, *P*, *V*, *WD*) was 1 min. In addition to the session-targeted MetPak Pro weather, there exists a permanent weather station located in the southern part of Polana Izerska (Fig. 1c). The station is owned and maintained by Świeradów Forest Inspectorate, and it measures numerous weather characteristics at the height of 2 m a.g.l. with a temporal resolution of 12 min.

The measurements of vertical structure of the near-ground atmosphere were performed using a mobile Doppler SODAR (SOnic Detection And Ranging), manufactured by ELAT. The instrument provided continuous information on vertical component of wind velocity (*Z*) in a 350-m profile (the effective height of the measurement is limited by the power of signal, 3.8 kHz) during the sessions. Due to the 15 m “start zone,” the real height of the profile is 365 m a.g.l. (15 m of “start zone” and 350 m of the entire profile). Vertical resolution of the measurements was of 2 m and time resolution was equal to 4 s. The precision of measurement of *Z* component was of 0.12 ms^−1^, with range measurements from −15 to 15 ms^−1^.

**Fig. 5:**
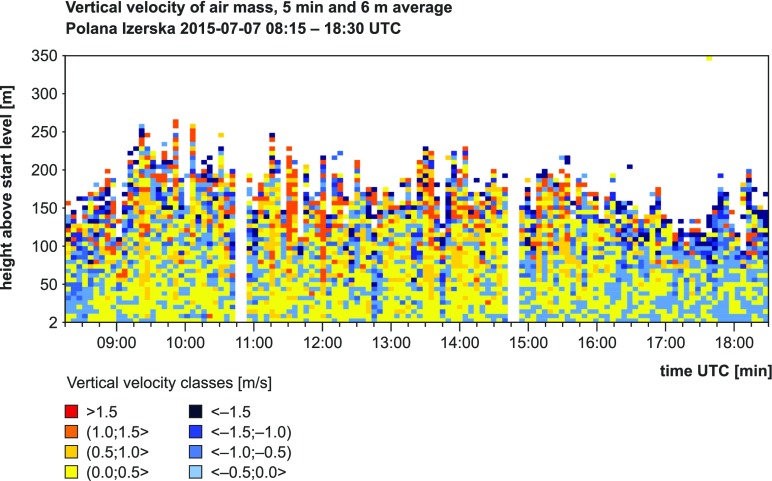
Averaged results of SODAR measurements of vertical wind velocity *Z* during the first day of the measurement campaign (07/07/2015, 08:15–18:30 UTC)

The change of general advection pattern observed during the measurement campaign had changed the airflow dynamics between the first and second day of the campaign. On 7 July 2015, during the entire measurement period, turbulent air movements of convective genesis were predominant. This was indicated by the observed convective *Cu* clouds development as well as confirmed by the registered SODAR data. Therefore, for most of the measurement period over Polana Izerska upward air movements dominated, which in total accounted for 59.5% cases and were most intensive between 9:00 and 15:00 UTC (Table 4, Fig. 5). In that time range, the upward movements accounted for more than 60% and mean hourly *Z* values for the whole SODAR measurement profile were above 0. In the evening (16:00–18:00 UTC), gradual blanking of convection was observed. As a consequence, the frequency of descending air movements increased and mean vertical air velocity (*Z*) was significantly below 0 (Table 4).

**Table 4:** Characteristics of horizontal (*V*) and vertical (*Z*) components of wind velocity in Polana Izerska during measurements campaign on 7–8 July 2015; *V* component—results from anemometer (10 m a.g.l.); *Z* component—results from SODAR measurements (averaged frequencies from whole vertical profile)

		Time UTC													
		06:00	07:00	08:00	09:00	10:00	11:00	12:00	13:00	14:00	15:00	16:00	17:00	18:00	All
07/07/2015	avg *V* [ms^−1^]	–	–	–	–	–	3.6	4.0	4.8	5.5	5.3	4.9	4.4	4.6	4.7
	max *V* [ms^−1^]	–	–	–	–	–	6.4	8.1	8.4	8.8	9.4	9.0	7.8	8.0	9.4
	avg *Z* [ms^−1^]	–	–	−0.23	0.11	0.04	0.19	0.06	0.03	0.16	0.08	−0.07	−0.37	−0.47	−0.04
	ascend. [%]	–	–	55.3	62.3	60.5	63.7	61.9	62.1	63.1	61.5	56.5	50.8	48.6	59.5
08/07/2015	avg *V* [ms^−1^]	5.0	5.7	5.7	5.5	6.3	6.7	6.3	5.1	4.2	–	–	–	–	5.7
	max V [ms^−1^]	9.8	9.9	10.4	9.3	10.4	11.3	10.6	8.1	7.7	–	–	–	–	11.3
	avg Z [ms^−1^]	−0.47	−0.54	−0.71	−0.69	−0.52	−0.66	−0.44	−0.51	−0.49	–	–	–	–	−0.56
	ascend. [%]	44.3	40.9	39.1	40.3	45.2	42	41	37.8	33.8	–	–	–	–	41.2

**Fig. 6:**
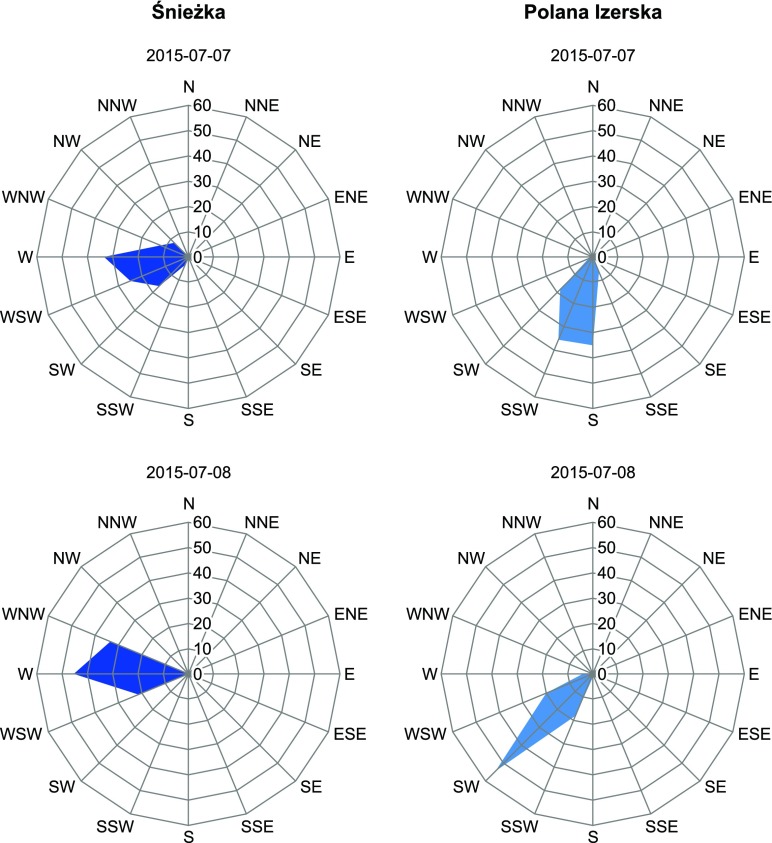
Frequency of wind direction at Mt. Śnieżka and in Polana Izerska during the measurement campaign

**Fig. 7:**
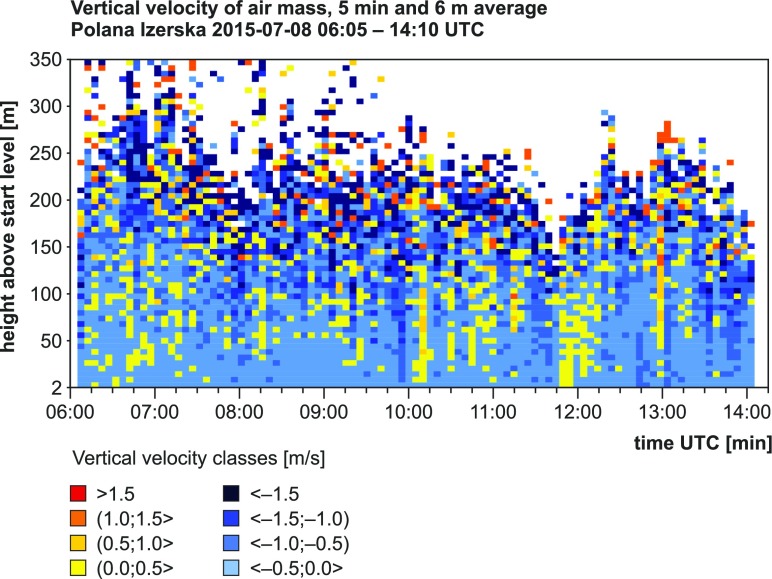
Averaged results of SODAR measurements of vertical velocity *Z* during the second day of the measurement campaign (08/07/2015, 06:05–14:10 UTC)

During the second day of measurements (8 July 2015), an increase in horizontal air velocity was observed, and the dominant (55%) airflow direction was SW (Fig. 6). The changes in synoptic situation were also reflected in vertical velocities measured using SODAR. Convection was reduced in comparison with the first day. This is confirmed both by visual cloudiness observations and SODAR data (Table 4, Fig. 7). Descending air movements were predominant (59% cases), and the mean hourly values of *Z* were below 0 and covered the range between −0.5 and −0.7 ms^−1^. The meteorological conditions described above indicate that on the first day of measurements, advective features of airflow were strongly disturbed by local conditions, connected mainly with convection development at the southern slopes of surrounding mountains during the day and accompanying intensification of turbulent air movements that resulted in higher variation of horizontal airflow directions and high share of ascending air movements. On the second day, due to limited convection, airflow occurred to be driven mainly by synoptic advective conditions.

The analysis of wind conditions in Polana Izerska should also consider that the observed wind direction differed from the general western air advection assumed for the entire area (Fig. 6). During the measurement period W–SSW and W–WNW wind directions were observed at Śnieżka, on the first and second day, respectively. In Polana Izerska, the wind direction was SSW–S during the first day and SW during the second day. The observed deviation of wind direction (45–65° towards the South) from the general direction was probably caused by local conditions connected with the role of terrain relief and land cover. The wind field deformation was caused by friction in the near ground level. However, it could also be driven by local deformation of airflow in the lowering of the Wysoki Grzbiet ridge, between Mt. Świeradowiec and Mt. Podmokła. In the case of the W–WNW advection, the pass may arrange the airflow, partially blocked from the east by Mt. Podmokła.

### Data processing methods

The meteorological measurements (wind speed and direction, air temperature, humidity) were gathered at 2.5 and 10 m a.g.l. It was therefore necessary to extrapolate the observed wind speed to the UAV height for comparison. There are several approaches for vertical extrapolation of wind speed, and the complete review is presented in a report that summarizes the COST Action 710: Vertical profiles of wind, temperature and turbulence (Cenedese et al. 1997) and Ebrink et al. (1997). For vertical extrapolation of wind speed, Cenedese et al. (1997) and Ebrink et al. (1997) recommend the use of Monin-Obukhov similarity theory (MOST) instead of simpler approaches based on, e.g., experimental power law profiles. In this study, we have followed the recommended approach based on MOST, and 2.5 and 10 m a.g.l. wind speed measurements were extrapolated to the actual height of the UAV. The reader is referred to Cenedese et al. (1997) for more details (see the equations 45 to 47). The MOST method is based on the following equation:

$$ u(z)=\frac{u_{\ast }}{\kappa}\left[\mathit{\ln}\frac{z}{z_0}-{\psi}_m\left(\frac{z}{L}\right)\right], $$

where *u*(*z*) is wind speed and height *z*, u_*_ is friction velocity, *κ* is the von Karman constant and *L* is the Monin-Obukhov length scale. *L* depends on vertical profile of temperature, and in the manuscript, the vertical profile is calculated from the measurements gathered at 2.5 and 10 m above ground level. Both u_*_ and *L* have been calculated using iterative method and dedicated scripts written in R software.

As the time steps of the UAVs and ground based measurements were different (and unevenly distributed for UAVs), the UAV height was taken for the time that was closest to the ground observation.

## Results

As noted earlier, two independent tests were performed, namely the reproducibility test and validation test. The reproducibility test aimed to check if different equipment (UAVs) may produce similar estimates of wind speed and wind direction. We considered two pairs of flights (swinglet CAM vs. eBee and Maja vs. eBee), and within each pair time gaps between landing of the first drone and launch of the other one were kept as minimal as possible to meet technical and logistics requirements. The validation test focused solely on wind speed and aimed at checking how wind speed estimates determined using the UAV measurements (Pitot tube + GNSS receiver) agree with data acquired using meteorological wind sensor installed on the 10-m mast. We selected three dissimilar flights so that each of the three UAVs was represented. The details of the flight configurations are presented in Subsection 3.1 and juxtaposed in Table 1.

### Reproducibility test

**Fig. 8:**
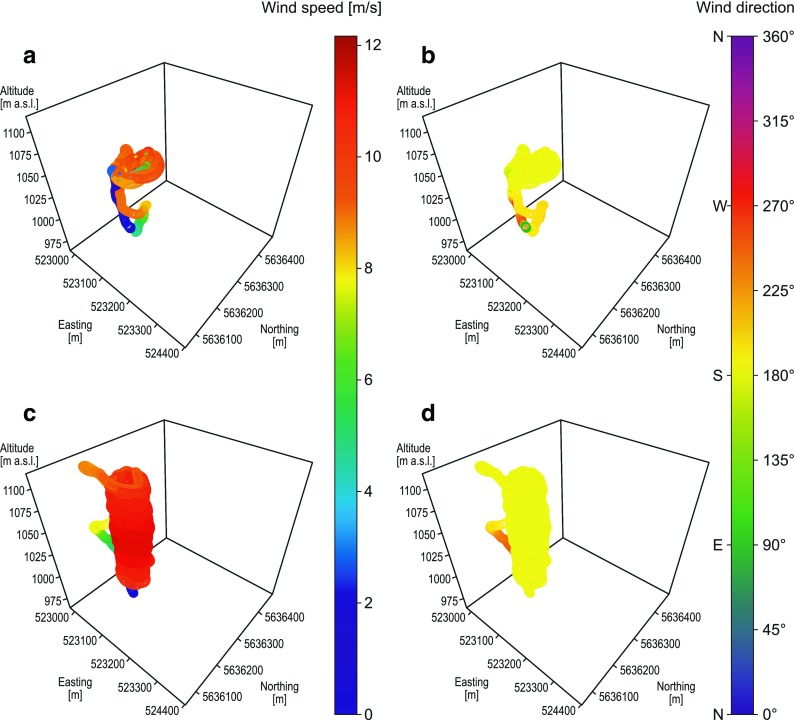
Wind characteristics along with spiral-like UAV trajectories on 7 July 2015. Wind speed estimated during flight no. 1 (swinglet CAM) at 18:23:26–18:28:59 UTC (**a**). Wind direction estimated during flight no. 1 (swinglet CAM) at 18:23:26–18:28:59 UTC (**b**). Wind speed estimated during flight no. 2 (eBee) at 19:01:52–19:18:04 UTC (**c**). Wind direction estimated during flight no. 2 (eBee) at 19:01:52–19:18:04 UTC (**d**)

Two flights with different drones (swinglet CAM and eBee) were performed in the evening on 7 July 2015. The first flight (swinglet CAM) began at 18:23 UTC and the second (eBee) about 38 min later. In general, estimated wind speeds for both drones were similar and varied between 8.0 and 11.5 ms^−1^ (Fig. 8a, c). The highest wind speeds were at about 1025–1050 m a.s.l. for both drones. Mean wind speeds were equal to 8.9 and 11.5 ms^−1^ at 1050 m a.s.l. with standard deviations equal to 1.7 and 0.9 ms^−1^, respectively, for swinglet CAM and eBee (Table 2). For both flights, wind direction was from the south sector, however it varied from SW to SE for the first flight and was mainly from S for the second flight (Fig. 8b, d). Mean wind directions at the height of 1050 m a.s.l. were very similar for swinglet CAM and eBee (180.9° and 179.4°, respectively). Slightly higher standard deviation was calculated for swinglet CAM (5.9°) than for eBee (3.7°).

**Fig. 9:**
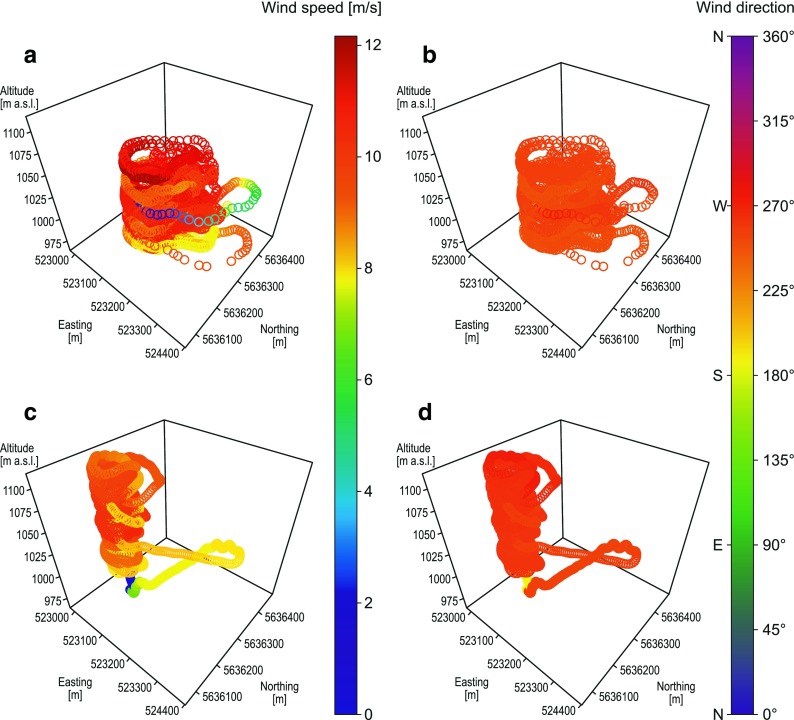
Wind characteristics along with spiral-like UAV trajectories on 8 July 2015. Wind speed estimated during flight no. 3 (Maja) at 07:59:51–08:18:28 UTC (**a**). Wind direction estimated during flight no. 3 (Maja) at 07:59:51–08:18:28 UTC (**b**). Wind speed estimated during flight no. 4 (eBee) at 08:24:37–08:34:55 UTC (**c**). Wind direction estimated during flight no. 4 (eBee) at 08:24:37–08:34:55 UTC (**d**)

Next two flights were performed on 8 July 2015 in the morning with Maja and eBee, respectively. The first flight began at 07:59 UTC and the second about 24 min later. In general, estimated wind speeds and wind directions for both flights were found to be similar (Fig. 9). Wind speeds varied between 8.0 and 12.0 ms^−1^, with the highest values calculated for Maja at the height of 1025–1050 m a.s.l. Mean wind speed at 1050 m a.s.l. was equal to 9.5 ms^−1^ for Maja and was approximately 1 ms^−1^ higher for Maja than for eBee (Table 2). Both drones showed a prevailing wind direction from WSW. Mean wind direction at 1050 m a.s.l. was of 239.9° for Maja and 244.9° for eBee with standard deviation of 8.7 and 9.6 ms^−1^, respectively.

### Validation test

**Fig. 10:**
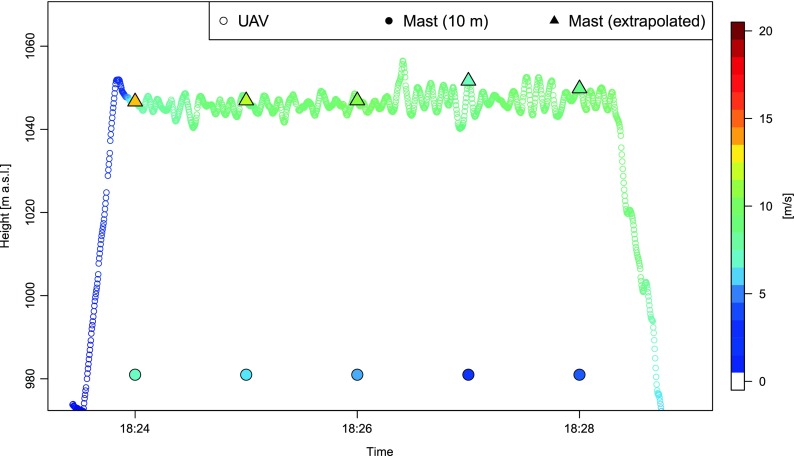
Wind speed estimated during flight no. 1 (swinglet CAM) on 7 July 2015 at 18:23:26–18:28:59 UTC as a function of time and height, against a background of altitude-corrected wind speed data measured at 10 m (mast)

Wind speeds estimated for the flight no. 1 (swinglet CAM) on 7 July 2015 in the evening were found to be lower than interpolated from measurements on the 10 m mast (Fig. 10). Underestimation was higher at the beginning of the flight (reached 5.0–6.0 ms^−1^) and decreased for the last 2 min of the flight to 1.0–2.0 ms^−1^.

**Fig. 11:**
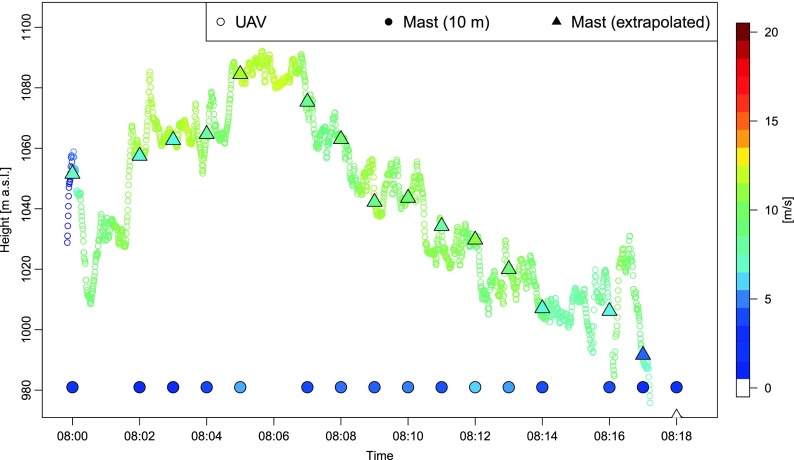
Wind speed estimated during flight no. 3 (Maja) on 8 July 2015 at 07:59:51–08:18:28 UTC as a function of time and height, against a background of altitude-corrected wind speed data measured at 10 m (mast)

Measured on the mast and interpolated to the appropriate height wind speeds were also underestimated by the estimations from Maja on 8 July 2015 in the morning (Fig. 11). The highest underestimation was observed at the beginning and at the end of the flight and reaches 3.0–4.0 ms^−1^. Better agreement was recorded in the middle part of the flight, with a perfect agreement noticed for several points of the period—e.g., 5th and 8th minute of the UAV mission.

**Fig. 12:**
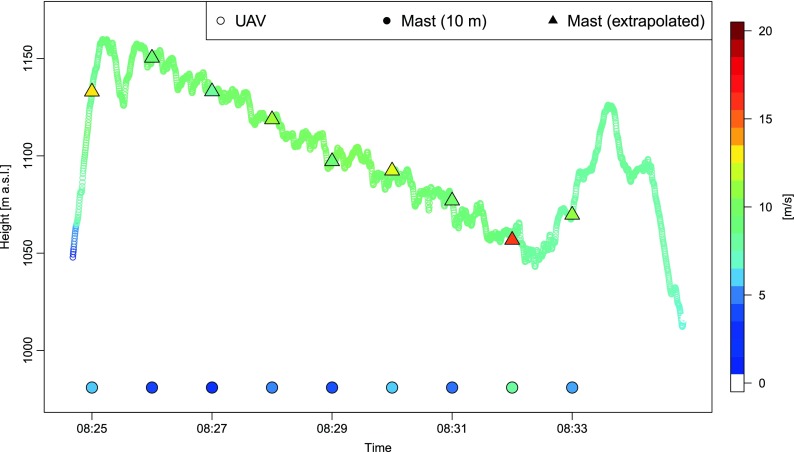
Wind speed estimated during flight no. 4 (eBee) on 8 July 2015 at 08:24:37–08:34:55 UTC as a function of time and height, against a background of altitude-corrected wind speed data measured at 10 m (mast)

A similar tendency was noticed for underestimation of measured wind speed on the mast by eBee (flight no. 4 on 8 July 2015 in the morning, Fig. 12). The underestimation was equal to 4.0 ms^−1^ at the beginning of the flight and decreased to 1–2 ms^−1^ in the middle of the flight. There was one outstanding value measured on the mast (8:31 UTC), which was highly underestimated by the drone.

Observed surface wind directions at 10 m a.g.l. were S–SSW and SW, while the UAVs systematically showed a difference during flight at 1025–1050 m a.s.l., with wind direction shifted more towards west direction.

### Discussion

Although there exist certain differences either between wind estimates based on different UAVs (reproducibility test) or between wind speed measured by the Pitot tube/GNSS receiver and wind estimates measured by the conventional meteorological instruments (validation test), our results may serve as a feasibility study that confirms the usefulness of the consumer-grade UAVs in meteorological applications. The approach is a reliable and relatively cheap source of meteorological information for the lower atmosphere, providing data at high spatial and temporal resolution that can be easily processed and used for various applications. The reproducibility test has shown that the different UAVs provide wind speed and wind direction data which are similar. The comparison with ground measurements extrapolated to UAV flight altitudes, carried out in frame of the validation test, shows that the UAV-based wind speed estimates are also in close agreement with professional meteorological measurements. The UAV agreeably showed a difference in wind direction during flight more than 100 m above the surface compared to surface observations. This difference agrees well with the development of the Ekman Spiral within the Ekman Layer where an acceleration of wind speed away from the surface layer (typically 30–50 m) cause a change in wind direction (e.g., Seinfeld and Pandis 2006).

Previous tests of the possible applications of UAVs in meteorology have been carried out by Reuder et al. (2009), Mayer et al. (2010), Reuder et al. (2012), Houston et al. (2012) and de Boer et al. (2016). These studies used only one UAV which usually was dedicated for meteorological measurements. In particular, de Boer et al. (2016) created the UAV system for measuring atmospheric radiation, atmospheric aerosol particle size distribution, and atmospheric thermodynamic state. They revealed a few problems in the initial phase of a flight, and they were identified as associated with orientation and rolling of a UAV. Houston et al. (2012) considered thunderstorm condition in the northeast Colorado and noticed an artificial increase in moisture and drop in temperature, also shortly after launch. Similarly, the experiments conducted by Reuder et al. (2009, 2012) for Svalbard showed that the biggest differences between wind speed profile obtained using a UAV and the Vaisala Radiosonde RS92 were recorded in first phase of the flight. Similar constraints for the initial and final phases of UAV flights are reported in our study which takes use of consumer-grade UAVs. However, the strength of the analysis presented in this paper resides in the use of three different drones which both confirmed reproducibility of UAV-based wind estimates and allowed independent verification of wind speed characteristics against wind estimates based on ground meteorological measurements.

The possibility to use consumer based UAVs as a device for estimating the changes in wind speed and direction has a substantial potential. A comparison of the surface wind speed and directions at the site as well as the drone observations with the three nearby WMO stations Liberec, Jelenia Góra and Śnieżka show that the local wind field varies substantially compared to the three stations. Substantial differences in wind directions, here reaching up to 65°, are expected for complex terrain and, in addition, may be caused by the differences in averaging time and distance between the WMO stations and Polana Izerska. Other more local estimates can therefore needed for such regions, e.g., in relation to air quality studies or in relation natural accidents such as forest fires. One approach is dedicated towers, but the UAVs offer an alternative that appear to be consistent with observations from high precision instruments, while they can be applied much faster and at a much lower cost compared to raising a tower. Furthermore, the UAVs can reach higher altitudes than towers and thereby provide a better estimate of the surface and Ekman layers. UAVs should therefore in certain situations (e.g., low cost or emergency situations) be the first choice of instrument compared to a traditional tower based instrumentation while they in other situations can complement observations from the towers.

## Conclusions

The results presented in the paper show that the consumer-grade UAVs may serve as a source of reliable meteorological information for various applications. The wind speed calculated with Pitot tube and GNSS sensors are in good agreement with meteorological observations. The following key findings can be inferred.

- Different UAVs equipped with the Pitot tube/GNSS receivers to measure wind speed and wind direction, both micro drones (swinglet CAM, eBee) and mini drone (Maja), acquire the wind estimates which are similar to each other.
- Wind speed estimates, based on measurements with the Pitot tubes/GNSS receivers installed onboard the above-mentioned three UAVs, agree with wind speed records measured by professional meteorological sensors installed at the 10-m mast (wind meters) and extrapolated to flight altitudes—this holds especially when the UAV is airborne (except initial and final phases of a mission).
- Wind vectors, derived by the UAV software using readings from the Pitot tube and GNSS receivers as input, were found to be agreed well between all UAVs.

Previous applications of the UAV meteorological data included for instance assimilation in mesoscale meteorological models and evaluation of the model results (Mayer et al. 2012; Jonassen et al. 2012). The growing number of photogrammetric commercial-grade drones which, according to the results of this paper, collect valuable data needed to estimate wind speed and wind direction may become an additional source of meteorological data to be assimilated to mesoscale meteorological models. This brings new potentials for improving skills of NWPs. Yet another group of potential applications of wind estimates calculated in real time onboard UAVs includes solutions targeted at environmental monitoring using both aerial images and meteorological data. An example of such applications is estimating snow water equivalent using snow depth map (based on aerial images) and meteorological data (based on Pitot tube/GNSS measurements), as exemplified by Miziński and Niedzielski (2017).
